# Getting vaccinated or not getting vaccinated? Different reasons for getting vaccinated against seasonal or pandemic influenza

**DOI:** 10.1186/1471-2458-13-1221

**Published:** 2013-12-21

**Authors:** Roberta Bonfiglioli, Michela Vignoli, Dina Guglielmi, Marco Depolo, Francesco Saverio Violante

**Affiliations:** 1 Occupational Medicine, Department of Medical and Surgical Sciences, Alma Mater Studiorum, University of Bologna, Via Pelagio Palagi 9, 40138 Bologna, Italy; 2 Department of Psychology, Alma Mater Studiorum, University of Bologna, Via Filippo Re 6, 40126 Bologna, Italy; 3 Department of Educational Sciences, Alma Mater Studiorum - University of Bologna, Via Filippo Re 6, 40126 Bologna, Italy

**Keywords:** Vaccine, Pandemic influenza, Seasonal influenza, HCWs

## Abstract

**Background:**

A large number of studies have investigated the motivation behind health care workers (HCWs) taking the influenza vaccine. But with the appearance of pandemic influenza, it became important to better analyse the reasons why workers get vaccinated against seasonal and/or pandemic influenza.

**Methods:**

Three main categories of reasons were identified with an Exploratory Factor Analysis. An analysis of variance (ANOVA) was used to verify the existence of differences between three categories of choices (taking of seasonal and pandemic vaccine, only the seasonal vaccine or none). In addition, a multinomial logistic regression analysis was performed to analyse the association between stated intentions and update of seasonal and pandemic vaccine. Questionnaires were returned from 168 HCWs (67.3% women).

**Results:**

The results showed that age and being well-informed about vaccination topics are the most important variables in determining the choice to take the vaccine.

**Conclusions:**

The results highlight the importance of enhancing education programs to improve awareness among HCWs concerning the benefits of taking the influenza vaccination, with particular attention paid to younger workers.

## Background

Health care workers (HCWs) are encouraged to get vaccinated every year against seasonal influenza. There are many ways to reduce influenza risk transmission, but the most effective remains the vaccine [1].

HCWs represent a strategic target for vaccination campaigns because they can transmit influenza viruses to patients, in whom the resulting infection can worsen said patients’ conditions. Decreasing the exposure of HCWs to the influenza virus through the vaccination of direct care providers can protect patients from serious consequences [2].

Furthermore, influenza affects between 5% and 10% of the population, and this has an effect on social costs because workers with influenza consult doctors and may be admitted to hospitals [3]. The Center for Disease Control and Prevention has advised vaccination for HCWs since 1981, and the World Health Organization (WHO) began supporting this practice in 2002. Despite this, the number of HCWs who get vaccinated has remained low (less than 25%). Italian data are even lower: 17.9% in 2010/2011 [4]. Regarding the pandemic virus, the national coverage among HCWs at the end of a vaccine campaign, based on doses administrated and on the number of people that got the vaccine in 2009/2010, was about 15% (this figure decreased in subsequent years) [5].

These data suggest that free vaccination has not been sufficient to increase the vaccination rate. It is thus important to understand the main reasons why HCWs get vaccinated in order to try to increase participation in vaccination campaigns.

Many studies have been conducted in the attempt to understand the characteristics of the people who decide to get vaccinated. Reviews and meta-analyses have investigated the choice to get vaccinated. For example, a recent meta-analysis [6] concluded that being male, being older than 40 years old, and being a doctor are demographic predictors that were positively associated with being vaccinated, while being a nurse was negatively associated with the choice to get vaccinated.

The situation becomes further complicated by the appearance of pandemic influenza, since people must make a choice about getting vaccinated against this type of influenza as well.

In fact, when in 2009 the A/H1N1 virus appeared, it became necessary to administer a dual vaccination against both seasonal influenza and pandemic influenza.

Some authors [7] found that the vaccination against the A-H1N1 virus was strongly associated with age, the perception that the pandemic vaccine was safe, and having received the seasonal influenza vaccine.

The results of a study conducted by To [8], which investigated the determinants of acceptance of pandemic influenza vaccination, highlighted that the main reason for getting vaccinated was self-protection, while the reasons for rejecting vaccination were possible side effects, ineffectiveness of the vaccine and the mild nature of the disease.

Another study conducted by Setbone and Raude [9] found that socio-cognitive factors that predict pandemic influenza vaccination were level of worry, risk perception and previous experience with vaccine against seasonal flu. Similar results were found by Hellyer and colleagues [10], who argued that the main reasons to take the pandemic vaccine were being worried about contracting A-H1N1 virus, about transmission, and also, following the advice of health authorities. On the contrary, the reasons to not take this kind of vaccine are personal choices, and being worried about side effects.

Therefore, we cannot be certain that the reasons that induce HCWs to get vaccinated are the same for both vaccines, pandemic and seasonal.

In fact, theories about behavioural choices maintain that there are a number of variables that affect the choosing process. One of the best known theories in the field of social psychology claims that there are many different aspects involved in the adoption of a behaviour.

The theory of reasoned action [11] maintains that a specific behaviour is the result of a two-way process where, on the one hand, we hold beliefs with respect to the consequences of a behaviour and the attitude toward this type of behaviour, and, on the other hand, we hold normative beliefs and subjective norms with respect to that type of behaviour.

This two-way process leads to the intention to implement the behaviour, and subsequently, to the actual behaviour itself.

This type of theory supports the concept that the process which induces people to choose to get vaccinated can vary depending on the vaccine; for example, beliefs regarding the consequences of implementing a behaviour can vary in relation to the two types of vaccinations.

In the literature, we found studies that analyse the difference between the reasons people decide to get vaccinated against seasonal influenza and against pandemic influenza.

As outlined before, these reasons could be influenced by demographic variables, such gender and age.

Therefore, we decided to investigate: (1) What are the reasons people get vaccinated against seasonal influenza or pandemic influenza, and are these similar or different?; and (2) When making a decision about whether or not to get vaccinated, what influence is exerted by important variables such as gender and age?

To answer these questions, we investigated a group of HCWs in a large university hospital.

## Methods

### Ethics statement

The Ethics Committee of the Sant’Orsola-Malpighi Hospital approved the study.

### Participants and procedures

The study was conducted between December 1, 2010, and January 11, 2011, in an Italian hospital. During the influenza season of 2010-2011, a total of 477 HCWs received the influenza vaccine, and some of them received the vaccine at the workplace, while they were performing their regular duties, due to a special program aimed at offering vaccinations on site. The program was set up by the hospital in order to promote and facilitate vaccination among HCWs. During the study period, all the unvaccinated HCWs who underwent periodic health surveillance, or visited the vaccination/biological risk clinic, were invited to participate in the study. All subjects received an information sheet, and their informed consent was obtained. According to Italian Legislation, vaccination is offered to HCWs free of charge. A total of 193 HCWs were invited, but 21 refused to participate. Thus, the final convenience sample was of 172 HCWs (response rate was 89.1%).

Each worker completed two questionnaires: one that measured the reasons why the worker decided whether or not to get vaccinated against seasonal influenza, and the other that measured the reasons why the workers decided whether or not to get vaccinated against pandemic influenza.

Only four workers were vaccinated against pandemic influenza, but did not take the seasonal vaccine, so they were excluded from the analysis (except for Exploratory Factor Analysis). Three groups were therefore formed: both vaccines (N = 82; Group A); seasonal only (N = 33; Group B); no vaccine (N = 53; Group C). Data were processed anonymously.

### Tools

A questionnaire was created specifically for this research project, consisting of ten items examining the reasons why the HCWs decided whether or not to get vaccinated against seasonal influenza and pandemic influenza. Items’ questionnaires were prepared by two researchers according to the variables most investigated in the literature. Three independent judges (researchers in the public health field), in two different sessions, assessed item contents with respect to the aim of the study. After a discussion, the judges agreed that all the items referred to possible reasons for taking, or not taking, the vaccination. The questionnaire was self-administered. All of the items were scored on a five-point scale, ranging from ‘1’ (totally disagree) to ‘5’ (totally agree).

### Data analysis

To analyse our research hypotheses, we conducted a series of different analyses which are described below. The software we used was SPSS, version 19.

#### Exploratory factor analysis

First of all, an exploratory factor analysis was performed on both questionnaires, analysing the factors of the observed variables, based on the covariance between the observed variables [12].

#### ANOVA and χ^2^

To analyse the differences between the HCWs’ choices (both vaccines, seasonal vaccine only, no vaccine) we conducted a two-way ANOVA. As explained before, the group related to the workers that only take the pandemic vaccine has been deleted. This type of analysis allows us to understand whether differences exist with regard to age in the factors that resulted from the factor analysis. A *χ*^2^ test was conducted to analyze gender differences. Performing ANOVA for the variables related to gender and age could help us to better control the effect of those two variables in the process of choosing to get vaccinated.

#### Multinomial logistic regression analysis

Logistic regression allows us to analyse the variables that are predictive of various types of behaviour, that is, three different groups (based on the choice regarding the vaccine). Only the variables that showed statistically significant differences among the groups (p < 0.05) were included in the model.

## Results

### Descriptive analyses

Due to the elimination of four of the subjects, there were 168 HCWs. Most of them (67.3%) were women. The average age was 43.28 years (st. dev. = 10.83). Doctors constituted 31%; 28.6% were nurses; 31% performed health activities, but not in contact with patients; and 9.5% were nurse assistants.

### Exploratory factor analysis

We conducted two different exploratory factor analyses for the questionnaire regarding vaccination against seasonal influenza, and for the questionnaire regarding pandemic influenza. We used the principal components extraction method and requested Varimax rotation.

The two factor analyses showed very similar results (Table 1). This confirms the factor structure of our questionnaire. The analyses highlighted three main factors regarding the reasons for vaccination. We called the first one ‘public-health’ reasons because it includes items such as, ‘*to guarantee the functionality of health services*’, and, ‘*due to a sense of civil, ethical, and professional responsibility*’. The second factor was labelled ‘personal/family’ reasons because it included two items: ‘*because I belong to a category at risk*’ and ‘*because I live with and/or I am close to family members who belong to one of the categories at risk*’. The last dimension was reasons regarding, ‘awareness of vaccine safety and side effects’. It included items regarding the quantity and quality of information that workers think they have, such as, ‘*because I think that vaccination is a safe practice*’ or ‘*because I have received sufficient information regarding the usefulness of vaccine*’.

**Table 1:** Rotated factor Matrix for both questionnaires (seasonal and pandemic influenza)

**Items**	**Seasonal vaccine**			**Pandemic vaccine**		
	**PH**	**PF**	**AW**	**PH**	**PF**	**AW**
To guarantee the functionality of health services	.850	.122	.007	.850	-.089	.003
In order to reduce the risk of infection and influenza virus circulation within the hospital	.823	-.049	.235	.797	-.083	.289
Due to a sense of civil, ethical, and professional responsibility	.771	.059	.177	.766	.125	-.007
To reduce healthcare organization costs	.758	.063	.108	.700	.153	.072
Because I have received sufficient information regarding the safety of vaccine	.022	.116	.886	-.009	-.109	.847
Because I have received sufficient information regarding the usefulness of vaccine	-.004	.117	.781	.015	.042	.785
Because I do not fear that the vaccine may be detrimental to my health	.280	-.102	.685	.182	-.161	.774
Because I think that vaccination is a safe practice	.308	-.045	.644	.219	.391	.472
Because I live with and/or I am close to family members who belong to a category at risk	.022	.814	.056	-.011	.854	-.009
Because I belong to a category at risk	.107	.806	.013	.052	.801	-.136

Note: Factor loadings of: PH = Public Health; PF = Personal/ Family; AW = Awareness of vaccine safety and side effects.

Following the exploratory factor analysis, we conducted the consistency measures.

The reliability scale ranged from 0.53 to 0.83. Except for the ‘personal/family’ reasons factor (only two items), all the alpha values exceeded the threshold of 0.70 [13].

Data in Table 2 show a good factorial structure.

**Table 2:** Scale and consistency measures

**Variables**	**Item**	**Seasonal influenza**			**Pandemic influenza**		
		**M**	**d.s.**	**α**	**M**	**d.s.**	**α**
Public Health	4	4.15	0.99	.828	4.20	.94	.790
Personal/Family	2	2.35	1.48	.529	2.66	1.61	.643
Awareness of vaccine safety and side effects	4	3.98	0.93	.769	3.73	1.02	.721

Note: M = mean; d.s. = Standard deviation; ***α*** = Cronbach’s ***α.***

### Univariate analyses

The three groups were compared on the basis of basic information variables and the factors used to analyse any differences among the groups. An analysis of the variance showed that significant differences exist among the three groups. These differences were also confirmed by Scheffè’s post-hoc test (Table 3).

**Table 3:** Univariate Analysis between the three different choice’s groups

	**Group A**	**Group B**	**Group C**	**Statistics**	**p**
	**Both vaccine**	**Seasonal only**	**No one**		
N	82	33	53		
Gender (Male)	31 (37.8%)	8 (24.2%)	16 (30.2%)	*χ*^2^ = 2.2	.334
Age	45.6 (11.1) §	43.9 (10)	39.2 (9.8) §	F = 6.1	.003
Seasonal vaccine					
PH	4.35 (.74) §	4.07 (.92)	3.88 (1.27) §	F = 3.9	.022
PF	2.30 (1.41)	1.97 (1.33)	2.66 (1.62)	F = 2.3	.100
AW	4.11 (.78)	4.00 (.90)	3.76 (1.13)	F = 2.3	.102
Pandemic vaccine					
PH	4.35 (.74)	4.01 (1.07)	4.07 (1.08)	F = 2.2	.109
PF	2.87 (1.38) +	3.14 (1.80) +	2.94 (1.70)	F = 4.6	.011
AW	4.09 (.80) + §	3.13 (1.02) +	3.54 (1.10) §	F = 13.6	.000

Note 1: Post hoc Scheffè; GroupA vs GroupB: +; GroupA vs GroupC: §.

Note 2: PH = Public Health; PF = Personal/ Family; AW = Awareness of vaccine safety and side effects.

Note 3: For the categorical variable “gender” the frequencies and percentages (in brackets) are provided. For all the other variables (continuous) the means and standard deviation (in brackets) are provided.

HCWs that did not take any vaccination were younger than those who took both vaccinations. Furthermore, the HCWs who took both vaccinations showed higher values regarding reasons of public health than those who chose not to get vaccinated. Also, the HCWs who decided to only get vaccinated against seasonal influenza showed higher values in reference to ‘personal/family’ reasons.

The largest differences among the groups were encountered in the factor of reasons regarding, ‘awareness of vaccine safety and side effects’ for the pandemic influenza vaccine. HCWs who took both vaccinations have higher values in this factor than those who only got the seasonal vaccination, or neither vaccination.

### Multinomial logistic regression analysis (nominal)

Multinomial logistic regression analysis was used to evaluate the strength of the relationship between the variables identified by the univariate analysis, and the fact of belonging to the group. Therefore, the following were included in the multivariate model: age, reasons of ‘public health’ with regard to the seasonal vaccine, ‘personal/family’ reasons with regard to the seasonal vaccine, ‘personal/family’ reasons and reasons regarding ‘awareness of vaccine safety and side effects’ for the pandemic vaccine. In Table 4 the whole Multinomial Logistic Regression Model is presented. The outcome variable consisted of the three groups of possible choices (both vaccines, only the seasonal vaccine, and no vaccine). The reference category was no vaccine. The model, which is a nominal one, was statistically significant (p < 0.00, LR *χ*^2^ = 48.22, log likelihood = 297.72).

**Table 4:** Whole model of the Multinomial Logistic Analysis

		**Exp (B)**	**95% Confidence interval for Exp (B)**	
			**Lower B.**	**Upper B.**
**Group A ** **Both vaccine**	Intercept			
	Age	1.060	1.022	1.100
	Seasonal PH	1.441	.960	2.164
	Pandemic PF	.732	.575	.933
	Pandemic AW	1.633	1.083	2.463
**Group B ** **Seasonal only**	Intercept			
	Age	1.044	1.000	1.090
	Seasonal PH	1.326	.844	2.081
	Pandemic PF	1.043	.791	1.374
	Pandemic AW	.612	.384	.975

Note 1: The reference category is: no vaccine uptaken (Group C).

Note 2: PH = Public Health; PF = Personal/ Family; AW = Awareness of vaccine safety and side effects.

We can see the significant results in Table 4. The predictors of the choice to get vaccinated against both types of influenza are age, ‘personal/family’ reasons and reasons regarding ‘awareness of vaccine safety and side effects’ for the pandemic influenza vaccine. With each additional year of age, the probability of getting both vaccinations increases by 6% in comparison to not getting either vaccination. Each increase of one point on the scale in reference to the reasons regarding ‘awareness of vaccine safety and side effects’ for the pandemic influenza vaccine increases the probability of getting vaccinated against both by 63.3% in comparison to not getting any vaccination.

An increase in ‘personal/family’ reasons with regard to the pandemic vaccine decreases the probability of getting vaccinated against both by 26.8%.

The only statistically significant predictor of deciding to get only the seasonal vaccination are the reasons related to ‘awareness of vaccine safety and side effects’ for the pandemic influenza vaccine: each increase of one point reduces the probability of getting vaccinated against the seasonal virus by 38.8%.

## Discussion

Our results make it possible to better understand HCWs’ choices regarding influenza vaccination.

First of all, it is necessary to point out that a very small group of workers (only 4, which corresponds to 2.3% of the original sample) decided to only get vaccinated against pandemic influenza. In other words, people get vaccinated against pandemic influenza primarily in addition to the seasonal vaccine. One possible explanation is that HCWs assigns less importance to the consequences of the A-H1N1 virus than the seasonal virus. This result is in line with previous research [7].

It has already been shown that age is an important variable in determining the choice of the vaccine [6]. As we can see, people who decided to get vaccinated were older than those who decided to not get vaccinated. Both national and regional legislation [14] encourages people over 65 years old to get vaccinated, but in this sample no worker was over 65 (Influenza Prevention and control, Italian Ministry of Health). However, if it is true that the probability of being affected by chronic disorders rises as age increases, in our sample we found no correlations between age and belonging to a category at risk, and living with, or being close to, family members who belong to a category at risk (all significant levels are between .372 and .961).

The multinomial logistic regression also confirmed that age is an important predictive factor.

We can assume that among HCWs, because of the type of job, and because of their seniority (several years spent in health care professions), older respondents could feel a stronger sense of belonging and, therefore, could more closely identify with the values of the public health service.

The ‘public health’ reasons for getting vaccinated against seasonal influenza have higher values among HCWs who received both vaccinations than those who did not get any vaccination. However, having a high level of agreement with ‘public health’ reasons was not strongly related with the decision to get both vaccinations, or only the seasonal vaccination.

Thus, we can state that knowledge is the key factor for the choice to uptake the seasonal and pandemic vaccine. HCWs are usually informed via a campaign by health departments about seasonal influenza vaccine: the mere communication about the importance of a vaccine does not, therefore, seem sufficient. In other words, workers are subjected to the same stimulus (a yearly vaccination campaign with the same content), and they tend to get used to them. We cannot say the same for pandemic influenza vaccine, as it is not an ordinary event. In this case, the stimulus is new and more prominent, so that detailed information about vaccination makes the difference in making the choice.

In addition, we can imagine that in that specific moment, when the mass media invested a large amount of time discussing pandemic influenza, the information related to getting vaccinated against pandemic influenza had the largest impact. This conclusion is in line with previous research [15], which found that the most common reasons underlying an unwillingness or hesitation to get the vaccine against pandemic influenza were the possible side effects and lack of comprehensive field evaluation before marketing.

In fact, the reasons regarding ‘awareness of vaccine safety and side effects’ on the pandemic influenza vaccine were determinant in predicting the vaccination choice. This is due to the fact that the reporting of a higher level of agreement in having received enough information has a positive effect on getting vaccinated against both types of viruses, and an opposite effect on getting vaccinated only against seasonal influenza (demonstrated by a fairly high odds ratio). Regarding pandemic vaccination, these results are in line with the study by Bouadma and colleagues [16], who found that the choices to take vaccines are self-centred sociocognitive dimensions, such as the self-perception of benefits of the recommended preventive health action and health motivation (i.e., the opinion regarding the likelihood of the vaccine to preserve health).

Therefore, we can conclude that the following reasons are the most important predictors for getting the vaccine: ‘awareness of vaccine safety and side effects’, regarding the pandemic influenza vaccine, or the assertion that the vaccination is a safe practice, or thinking that a sufficient amount of information has been gathered regarding the usefulness of the vaccine. A recent review [17] regarding the influenza vaccine and HCWs also found that the lack of knowledge, or wrong beliefs in the efficacy of the vaccine and fear of side effects, could be determining factors in making the choice to get the influenza vaccine, or not.

For these reasons, it seems necessary that public health institutions that plan the information campaigns on vaccinations take these factors into consideration, especially if a new type of vaccine should be administered to the HCWs. Some authors [18] suggest that for this kind of campaign to be effective, it should be based on strategies of education and promotion of vaccination, as well as on easier access to the vaccine. Our results are in line with what is stated above; they suggest, in addition, that this kind of campaign should also be focused on the involvement of HCW participation in order to be effective and increase vaccination rates. The mere exposure to information, without assimilation within a personal set of knowledge and beliefs, is not sufficient.

These strategies could be more effective for HCWs with respect to other workers or persons because of their greater awareness of health topics. HCWs could benefit from specific training, with more detailed and comprehensive information regarding both the usefulness and the side effects of vaccinations. In literature, we could find some models that could help build an understanding of how to design effective campaigns or trainings. For example, in a recent study of Prati, Pietrantoni and Zani [19], the Extended Parallel Process Model (EPPM) [20] was analysed to better understand the persuasive messages concerning the influenza vaccination. This theory claims that fear appeals are likely to produce threat appraisal, and the appraisal of efficacy of the recommended response and self-efficacy. In addition to this, the theory postulates that high threats elicit negative emotions that, in turn, lead people to begin the efficacy appraisal. According to this, if the threat is perceived as low, people are not motivated enough to appraise the message and the efficacy. The results of this study show that narrative communication (e.g., use of anecdotes, testimonials, and stories) constitutes a more comprehensible and believable pedagogical communication. The authors found no relationship with the intention to vaccine, but this is likely due to the fact that the study was based on a telephone survey, and not a ‘real’ setting. Although the study was conducted with a sample of older people (more than 65), it is the first one that used the EPPM model to analyse the efficacy of messages to promote influenza vaccination.

Some limitations of this study have to be acknowledged. First of all, the sample is quite small, so future research should consider a larger number of workers involved in the study. In addition, we should also consider the fact that both scales have a limited number of items. Furthermore, the sample of our study were recruited at the vaccination/biological risk clinic, and this could produce bias.

Moreover, future research is needed about the efficacy of interventions aimed at improving vaccine-taking behaviour. This could be helpful for an in-depth analysis of the reasons that lead to take, or not take, the vaccine, and also in terms of excessive time and money consumption reasons.

## Conclusions

The results of this study showed that among HCWs the perception of having been adequately informed about the usefulness of vaccination, as well as about vaccine safety, is an extremely important factor which influences the choice to take the influenza vaccination.

Hence, this study contributes to better define the main contents that have to be included in a training or education program to make sure that workers are more aware about their vaccination choices. In designing this kind of intervention, it is important to take into account that younger and older worker have different attitudes and beliefs regarding the reasons to take the vaccine.

## Abbreviations

HCWs: Health care workers; SPSS: Statistical Package for Social Science; ANOVA: Analysis of variance.

## Competing interests

The authors declare that they have no competing interests.

## Authors’ contributions

All authors discussed the methodological biases of the study providing an important contribution. Besides this, all of the authors discussed the structure and contents of the study. RB and FSV performed the experiments, MV and DG analysed the data, MV, MD and DG contributed to the analysis tools. All authors read and approved the final manuscript.

## Authors’ information

The authors belong to research teams that have been operating for a long time within the field of occupational health, with special attention towards the knowledge transfer from academic research to professional interventions.

## Pre-publication history

The pre-publication history for this paper can be accessed here:

http://www.biomedcentral.com/1471-2458/13/1221/prepub
